# Drivers and levers of the double burden of malnutrition in Cape Town, South Africa: insights from in-depth interviews with multi-sectoral stakeholders

**DOI:** 10.1186/s12889-025-24210-0

**Published:** 2025-08-29

**Authors:** Nicole Holliday, Mulalo Kenneth Muhali, Martina Lembani, Hlolisiso Nonkeneza, Maxwell Feni, Zandile June-Rose Mchiza, Jillian Hill, Carmen Klinger, Eva A. Rehfuess, Peter von Philipsborn, Peter Delobelle

**Affiliations:** 1 Institute for Medical Information Processing, Biometry and Epidemiology (IBE), Chair of Public Health and Health Services Research, Faculty of Medicine, LMU Munich, Munich, Germany; 2 Pettenkofer School of Public Health, Munich, Germany; 3 School of Public Health, University of the Western Cape, Belville, South Africa; 4 Non-Communicable Diseases Research Unit, South African Medical Research Council, Tygerberg, South Africa; 5 Chronic Diseases Initiative for Africa, University of Cape Town, Cape Town, South Africa; 6 Department of Public Health, Vrije Universiteit Brussel, Brussels, Belgium

**Keywords:** Food environment, Enabling environment, Unhealthy diets, Public health, Complex systems, Health policy, Double burden of malnutrition

## Abstract

**Background:**

South Africa faces a high burden of malnutrition, including undernutrition, overweight, obesity, and diet-related non-communicable diseases. This coexistence and interaction of multiple forms of malnutrition within individuals and communities across the life-course is referred to as the double burden of malnutrition (DBM) and has complex, interrelated causes that need to be concurrently addressed. This qualitative study explored the drivers and potential leverage points of the DBM at individual, household, community, and (local) policy level in the Cape Town Metropolitan region.

**Methods:**

From November 2023 to April 2024, 35 in-depth, semi-structured interviews were conducted with community health workers (CHWs) and CHW coordinators, researchers, civil society representatives, and government employees. Interviews were conducted in isiXhosa or English, and transcribed and translated into English where necessary. Coding and analysis drew from grounded theory and complex systems thinking.

**Results:**

Across the individual, household, community, and local policy level, four central drivers of the DBM emerged: financial constraints, other resource constraints, food and nutrition literacy, and food quality of informal food business. At the individual and household level (micro-level), two additional barriers were identified: abuse of alcohol and other drugs and a lack of individual accountability. At the community and local policy level (meso-level), additional challenges included the power of the formal food industry, political inertia, and a siloed government approach. Leverage points at the micro-level included government social support programs and food gardens. At the meso-level, leverage points included an emphasis on the first 1000 days of life, food sensitive urban planning, strengthening networks, and adopting a systems response.

**Conclusions:**

This study revealed drivers of the DBM at the micro- and meso-level in Cape Town, as well as potential leverage points. By understanding the lived realities of those experiencing and working with the DBM, researchers can better understand the interconnected drivers and how these drivers manifest in everyday life. Local solutions to address the complex issue of the DBM require multi-sectoral stakeholder perspectives.

**Supplementary Information:**

The online version contains supplementary material available at 10.1186/s12889-025-24210-0.

## Introduction

For nearly twenty years, the prevalence of stunting among children under 5 years has remained virtually stagnant in South Africa, standing at 27% according to the 2016 national health survey [1]. Over roughly the same period, the prevalence of overweight and obesity among children and adults has increased, reaching 68% prevalence among adult women and 31% among adult men in 2016, and 14% among children aged 6–14 years in 2012 (latest national counts) [2, 3]. Diet-related non-communicable diseases (DR-NCDs) have likewise increased and the prevalence of hypertension has nearly doubled from 25% among adult women and 23% among men in 1998, to 46% and 44%, respectively, in 2016 [4]. While the country has implemented a range of nutrition policies in the past twenty years, it is clear the double burden of malnutrition (DBM), defined as the coexistence and interaction of undernutrition (stunting, wasting, underweight, micronutrient deficiencies) and overweight, obesity, or DR-NCDs, remains a prominent issue [5]. The DBM extends from urban to rural areas and affects individuals, households, and populations across the life-course [5].

In the Cape Town Metropolitan region (Cape Town hereafter), the second most populous city in South Africa, food insecurity is a persistent problem across income levels. In 2013 (the latest available survey data from the African Food Security Urban Networks), 58% of the 2,500 surveyed households in Cape Town experienced food insecurity, with 72% of low-income households reporting food insecurity [6]. As noted by Battersby et al. [6], food insecurity encompasses not only hunger, but also “long-term consumption of a limited variety of foods,” and can be described less as an issue of food availability, and more so as an issue of having “the economic and physical resources to access enough of the right kind of food” [6]. Understood in this way, food insecurity is linked not only to undernutrition and micronutrient deficiencies, but also to overweight and obesity, due to the consumption of low-diversity, energy-dense, and nutrient-poor foods.

Food insecurity in Cape Town further reflects a set of interrelated challenges related to, among others, multidimensional inequality and poverty; increasing urbanization, partly driven by international and internal migration, that has not been sustainably managed [7]; high levels of unemployment particularly among those living in informal settlements and townships (i.e., “urban, residential areas that during Apartheid were reserved for non-whites who lived near or worked in areas that were designated ‘white only’”) [8, 9]; inadequate access to healthcare and to water, sanitation, and hygiene infrastructure within informal settlements and townships [10]; and continued social exclusion of Black Africans and people of mixed ancestry (‘Coloureds’ in the official South African terminology) [11].

Progressive approaches to addressing food insecurity have been implemented in Cape Town in recent years. In 2007, it was the first city in South Africa to create an Urban Agriculture Policy [4]. In 2021, following a water crisis in 2018 and the COVID-19 pandemic, Cape Town also introduced its Food Systems Programme (FSP), a strategy that “aims to create a food system that can produce enough affordable, nutritious, sustainably grown food and can handle shocks” by integrating food system elements into relevant plans, policies, strategies, and by-laws [12]. The current FSP Working Group is led by academics and provincial government representatives [12]. The development of the FSP drew on systems thinking (also called system dynamics or complex systems approach), an approach to “model process structures and analyze their behavior through the investigation of how resources flow, accumulate and interact in the system, over time, in dynamic interdependent feedback loops” [13]. Systems thinking is used to develop a comprehensive, integrated understanding of complex adaptive systems, i.e., systems characterized by a range of interrelated factors and their dynamic relationships, including feedback loops, non-linearity, and emergence [14]. The DBM can be considered both an element and an outcome of a complex adaptive system [15]. As such, a systems thinking approach allows for a more comprehensive examination of the DBM within the layers of the wider food system, the food environment, and other structural issues like poverty and inequality.

While Cape Town is making efforts to address urban food insecurity, progress in reducing the DBM is limited, and food insecurity continues to impact the quality of life among residents of all backgrounds. This study forms part of the research project Food Environments in Africa: Addressing Malnutrition using a Syndemics Approach’ (FoodSAMSA), which aims to address the DBM and its determinants in South Africa, and explore interventions at macro-(policy), meso-(community) and micro-(interpersonal) levels. In addition to producing stand-alone insights, this study was conducted to inform group model building to develop complex systems maps of the drivers and levers of the DBM, with results described elsewhere (manuscript under preparation; [16]).

This qualitative study, using a complex systems lens and drawing on grounded theory, sought to better understand perspectives on challenges and possibilities to addressing the DBM from a diversity of multi-sectoral stakeholders, involving affected community members, non-governmental organizations (NGOs), researchers, and government employees.

## Methods

### Overview

This is a qualitative study based on semi-structured interviews with a broad range of stakeholders representing the micro- (individual and household) and meso- (community and local policy) level.

The micro-level included community health workers (CHWs), outreach team leaders (OTLs) of the community oriented primary care teams, and their coordinators. CHWs are trained lay health workers that provide the “first line of support” to communities for health needs and connections to social services [17]. OTLs ensure that the CHWs are supported and supervised and that their work is connected to service delivery targets [17]. These stakeholders were asked to offer their understanding of drivers and levers of the DBM in their own lives as well as in their capacity as representatives from their communities.

The meso-level encompassed researchers with a background in nutrition, food security, urban studies, or food systems policy; employees of food and nutrition NGOs; and food system and nutrition-related policymakers from the local level (provincial and city government). Meso-level stakeholders were asked to provide reflections on their understanding of drivers and levers at both the local/community level (e.g., in townships) and at the Metropolitan/policy level.

### Study setting

Located in the Western Cape and bordered by the Atlantic Ocean, Cape Town has a population of nearly 4.7 million people and is the second largest economic center in South Africa [18]. As in other parts of South Africa, the city is characterized by legacies of Apartheid, including stark wealth, income, and housing inequalities [19]. In 2020, 28% of households were living in poverty (defined as a monthly earning of 3,500 Rand or less [approx. 480 USD Purchasing Power Parity (PPP) as of March 2025]), with Black Africans disproportionately affected [8]. In 2021, the city’s unemployment rate was 30% with one-quarter of all youth (15–24 years) not involved in education, employment, or training [8]. Crime is a significant issue, and murder rates (67 per 100,000 in 2021/2022) are higher than the national average [8]. Cape Town is considered to be South Africa’s most segregated city with affluent neighborhoods contrasted by crowded informal settlements and underdeveloped townships [9, 19]. Many of those living in townships and informal settlements rely on the informal economy for survival, which also usually provides lower wages than formal employment [20]. Those employed in the formal sector often need to commute to the economic hubs of Cape Town, which are not found within the residential urban sprawl of the townships but rather in the wealthier suburbs and city center. The three townships included in our work were Nyanga, Gugulethu, and Kensington.

Nyanga is a predominantly Black African (99%) neighborhood with only 55% of the labor force (15–64 years) employed (as of 2011, latest available counts) [21]. The average household size is 3.6 with 74% of households earning a monthly income of 3,200 Rand or less [approx. 440 USD PPP as of March 2025] (as of 2011, latest available counts) [21]. Nyanga is particularly burdened with crime and the Nyanga police precinct has the fifth highest number of reported crimes in Cape Town [8]. Gugulethu is another predominantly Black African township (99%) with 60% of the labor force employed, an average household size of 3.3 people, and with 71% of households earning a monthly income of 3,200 Rand or less (as of 2011, latest available counts) [22]. Kensington is slightly different, with a mostly (91%) multiracial neighborhood (‘Coloured’/mixed-ancestry in the official South African terminology) with 86% of the labor force employed [23]. The average household size is 4.2 people and 33% of households earn 3,200 Rand or less per month (as of 2011, latest available counts) [23].

### Participant selection & recruitment

Participants for the micro-level interviews were recruited purposively from three community health NGOs that are partners in the FoodSAMSA project, including the ANOVA Health Institute (Gugulethu), Etafeni (Nyanga), and Kheth’Impilo (Kensington). After receiving approval from the NGO administrators, we asked CHW coordinators from each of the three sites to recommend CHWs who either spoke isiXhosa, English, or Afrikaans (the three languages most common in the areas and for which we had capable interviewers) and who were willing to be interviewed. NGOs were offered monetary compensation to cover the time effort of the involved CHWs.

Participants for the meso-level interviews were selected based on background and expertise in nutrition, food systems, urban studies, or food security and policy in the Western Cape. We used purposive sampling facilitated by a researcher with expertise in food systems research in South Africa (FK, listed in the acknowledgements). Participants were recruited via email and phone and did not receive a compensation.

For the micro-level, we planned to include at least five participants from each study site to account for demographic differences between the townships. Data analysis was done concurrently with data collection and by the third study site, theoretical saturation (i.e., no further insights regarding the emerging theory) was reached, with no major differences in themes noted between study sites [24]. For the meso-level, a sample size was not defined and recruitment for the interviews ended after theoretical saturation was reached.

### Data collection & analysis

Interviews were conducted between November 2023 and April 2024 by trained researchers using a semi-structured interview guide (see supplementary material). Interviewer training included discussions of research ethics, interview techniques, and decolonial approaches to qualitative research. For the meso-level, two members of the research team conducted the interviews (NH & PD), one of whom is a 20 + year resident of South Africa from Europe and the other a researcher from the United States on a three-month field visit in Cape Town. The interviews were held online (via Zoom) and in English. For the micro-level, four members of the research team conducted the interviews (MF, MMK, HN, NH), three of whom are from South Africa and fluent in isiXhosa, whilst the fourth is from the United States. These interviews were conducted in English or isiXhosa and conducted in private to protect confidentiality at community health centers in Nyanga, Gugulethu, and Kensington.

Written informed consent and willingness to be audiotaped were obtained from all participants. Interviews were recorded using either portable audio recorders for the in-person interviews or Zoom recordings for online interviews. The interviews were transcribed verbatim and translated into English, when necessary, by two native isiXhosa speakers. Analysis drew from complex systems thinking and constructivist grounded theory (originally described by Corbin & Strauss and expanded upon by Charmaz) [25–27]. These approaches were selected in recognition of the complexity of examining the DBM and to center lived experiences. Grounded theory has often been used in decolonial approaches to health research as it emphasizes the active and subjective role of the researcher in the theory development process and aims to allow theories to emerge inductively from the data [28, 29].

Inductive coding using the constant comparative method was done by the primary author (NH) throughout data collection, starting with open coding and followed by sorting of codes into emergent categories. A codebook was then developed by the primary author. Additional team members (PD, CK, HN, ML, JH, ZJM, MMK, MF) conducted simultaneous group coding using one to two transcripts per researcher and coding topics (independent of the codebook) related to the major drivers of the DBM and potential leverage points. The codes were discussed within the team and condensed into topic variables based on similar themes. Group coding was conducted for both micro- and meso-level interviews. The codebook was then updated by the primary author (NH) and transcripts re-coded as necessary. MAXQDA24 was used to facilitate coding and analysis [30].

### Ethical considerations & reflexivity

This study received approval from the ethics review board of the University of the Western Cape (ethics reference number BM22/2/7), the University of Cape Town (567/2022), and Ludwig-Maximilians-Universität München (LMU Munich) (reference number 22–0505). All study participants were informed of their rights and provided written consent before participation.

The research team included researchers from institutions in South Africa and Germany and reflected both insider and outsider perspectives. Several team members had training and experience in qualitative research, and several had training in decolonial approaches to qualitative research. The team was diverse in gender and ethnicity and the primary author spent three months in Cape Town during data collection allowing a deeper understanding of the context. Reflective debriefings were conducted by the primary author among the interviewers (with the exception of five interviews done in Nyanga). In some cases, we do not report the precise occupation of interviewees in the quotations below to protect their identity.

## Results

### Overview of findings

Thirty-five interviews were conducted, 14 with stakeholders at the meso-level and 21 with stakeholders at the micro-level. Interview duration ranged from 30 to 100 min (median length 51 min). Ten interviews were held in isiXhosa, the rest in English. Interviewees ranged in age from 29 to 68 years (median age 48 years). Further characteristics of the interviewees are presented in Table 1.

**Table 1:** Characteristics of interviewees

	Micro-Level	Meso-Level
Gender	Woman (18) Man (3)	Woman (8) Man (6)
Employment position	CHW (13) Outreach Team Leader (6) CHW Coordinator (2)	Civil society (1) Private business (1) City of Cape Town employee (3) Western Cape government employee (3) Academic (5) CHW administrator (1)

*CHW*: Community Health Worker

**Fig. 1:**
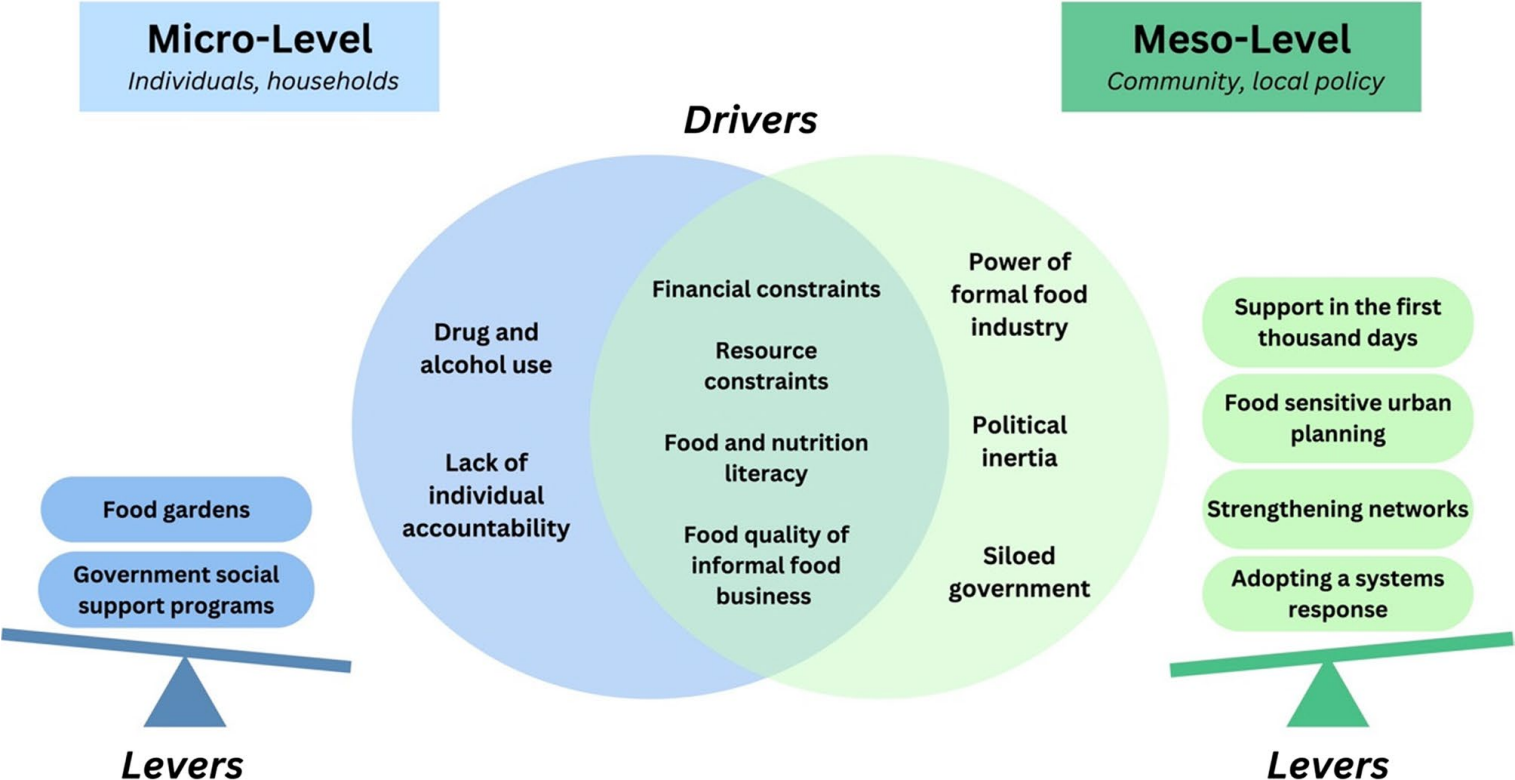
Main drivers and levers of the DBM at micro- and meso-level

Several themes emerged across both stakeholder groups; in addition, there were drivers and leverage points unique to each stakeholder group (see Fig. 1).

### Shared micro- & meso-level drivers

Across micro- and meso-level study participants, parallel drivers of the DBM were found— financial constraints, resource constraints, food and nutrition literacy, and food quality of informal food businesses.

### Financial constraints

The most prominent driver of the DBM expressed by both participant groups was poverty. Micro- and meso-level participants described how the historical inequality, high unemployment rates among township populations, and the lack of meaningful social welfare limited people’s ability to choose or access healthy foods. CHWs were frustrated by the fact that while a large part of their role is to educate people on healthy diets, for many households, a healthy diet is not financially feasible.

“We tell people about the healthy diets, and we go to the houses and talk. And then a lot of them are saying, ‘uhm, it’s good for you to say, to talk about the healthy food, but not for us … The only thing is money. To eat good and healthy food, it costs money.’” -CHW, Kensington.

For many households, nutrition is not prioritized due to competing resource demands; rent, school fees, and debts were all noted as higher priorities in the monthly expenses. As one meso-level stakeholder (food systems researcher) described, “…stopping the hunger today is the priority. Nutrition is something for tomorrow; if tomorrow ever arrives.”

As well as noting the effects of individual-level poverty, meso-level participants described a lack of funding for nutrition-related programs at the city and provincial level as another issue.

### Resource constraints

Closely related to the issue of financial constraints was the theme of resource constraints beyond income, including energy/electricity, cold storage for food, cooking appliances, and time scarcity at individual and household level. Both micro- and meso-level participants noted that township households have limited means of safely storing and preparing foods and find it cheaper to buy take-away food or small food packages at informal food businesses (which may be characterized by lower food quality and safety) than to shop for food in bulk. To accommodate the lack of electricity and time, it is also easier to buy easy-to-prepare food as people face long commute times to work and stores, and households are often single-headed or include multiple people working at the same time (e.g., less time for one parent to prepare meals for children). Participants mentioned the planning effort involved in preparing meals at home.

“Time also plays a part there. I’m coming up, I’m late from work. I didn’t plan for this so down to the Braai [barbecue] shop, buy whatever you can get. That’s our supper. So, planning plays a big role for people to eat healthy.” -OTL, Kensington.

Operating at the local policy (meso) level, the issue of constrained resources was also expressed in terms of personnel, time, and capacity. One of the political challenges identified was sustaining commitment and action to enact change in the long term, particularly in what participants described as a ‘multi-crisis’ and under-resourced governing environment. Meso-level participants discussed how government employees manage multiple projects and mandates and are often unable to fully interact with the affected communities. Concurrently, community members also manage multiple issues and have limited capacity to engage with new programs and policies brought by the government, particularly as administrations and policies change. While there is goodwill from many stakeholders—government employees, communities, NGOs—it is challenging to sustain energy for change while managing low budgets and capacities. One government employee described how individuals in resource-limited communities juggle multiple roles:

“And almost by definition, the spaces where you’ve got very deep pockets of food insecurity tend to be very resource-depleted… there isn’t a local government person. Their, like, dog catcher is the same person who’s also, like, the head of the fire department, who’s also now in charge of social development. And their, kind of, their eyes roll over, like glaze over when you try and tell them, ‘OK, well, now we’re gonna start reaching into your planning system to change the way the food flows in your space.’ They’re just like, please, I’m just trying to get to tomorrow.”

-Government employee, Western Cape Government.

### Food and nutrition literacy

Across the micro- and meso-level, food and nutrition literacy was discussed as an important driver of nutrition. Among CHWs, educating community members about nutrition and good health is a primary component of the job, and most believe that education is critical to improving individual health. However, the CHWs had differing perspectives on enablers to follow through on nutrition advice and education. For some, whether someone heeds the nutritional advice depends on their individual willingness and motivation, whereas for others, there is recognition that implementing the knowledge depends on financial abilities (linking back to the issue of income poverty).

“Actually, when you talk about that to me, it’s a personal choice to me. Because you can only teach people about good nutrition, but it’s up to them what they go for when they want to eat.” -CHW coordinator, Kensington.

“I think the education is helping them to know how to eat, but the problem is they will tell you, how are they going to get that healthy food? Because they don’t have money. The education is there. Every time they get education about nutrition, but the only thing they will tell you is how are they going to get that good nutrition?”

-OTL, Gugulethu.

Participants from the meso-level also stressed the importance of food and nutrition literacy for the general population as well as for policymakers to craft more nutrition-sensitive policies. As noted by some of the CHWs, meso-level participants described education as a necessary, but not sufficient component of behavior change to improve diets, with participants citing structural barriers like income and resource poverty.

“And so having the knowledge or the awareness, that’s only one side of it. So, there’s a lot to do with what people have access to in terms of information, what they are used to, their cultural implications of that. And a great deal in terms of their wherewithal, what they’re able to afford… So, education or awareness or knowledge is only one part of it, and that in itself is affected by a large number of dynamics. But for me, it’s whether that in fact has a real meaning or traction in a space that perhaps is not conducive to healthy eating.” -Food security researcher.

### Food quality of informal food businesses

Participants from micro- and meso-level described the important role of informal food business in the nutrition space in Cape Town, particularly spaza shops (informal convenience stores usually run by immigrants) and street food vendors (temporary, informal stalls on the side of the road that sell fresh foods, packaged food, and/or prepared foods like barbecued meat).

These informal businesses were reported to be more convenient, accessible, and affordable for township communities and play an important role in supporting the informal economy. However, poor food quality at informal shops was described as a contributor to the DBM, particularly regarding the high saturated fat content of meats, the predominance of processed foods, and issues of food safety.

“There’s a shop now near [location] that was closed on Friday because they sell expired stuff. So, you have to check. That’s why I don’t like going to spaza shops. I’d rather prefer to go to Spar [i.e., a formal supermarket] or the shop here opposite because I know they get fresh stuff in there every day. But I don’t trust the shops near me.”

-Micro-level actor, Kensington.

Meso-level participants agreed that informal food business plays a crucial role in the food system and needs to be involved in urban food planning to address the DBM. They acknowledged current limitations of the informal food business and suggested that health, social, and economic elements need to be taken into account, including food safety, economic trade-offs (e.g., stands selling processed, packaged foods are able to make more profit than those selling fresh produce), and spatial planning. Participants suggested city and provincial governments take a more active role in supporting and regulating informal food business.

“…I do think that firstly, far greater recognition of the informal sector, far greater acknowledgement of the benefit that they’re providing and so support for that benefit in terms of sanitation, access to infrastructure, etc. And it doesn’t mean excessive regulation. It means a slightly different approach to how they engage the informal sector.” -Food systems researcher.

Meso-level participants also described how the formal food industry (i.e., large, corporate actors) has more recently recognized these informal shops as a means to tap into new markets and are increasingly establishing more structured deals on promoting certain products. While this provides a new level of accessibility for township populations (e.g., selling smaller packets of brand products), it also contributes to saturating the market with more processed products.

### Micro-level drivers

Micro-level participants mentioned some specific drivers related to the DBM, including the influence of alcohol and other drug use and a lack of individual accountability for nutrition.

### Alcohol and drug use

According to the CHWs and their coordinators, drug and alcohol use constitute significant barriers to good nutrition in their communities. Interviewees reported that some individuals use welfare payments (called 'social grants' in South Africa) to buy alcohol and other drugs at the expense of other commodities, including food. In addition, alcohol and drug use hampers the uptake of nutrition and health education messages provided by CHWs and contributes to crime and ‘gangsterism.’

“Another challenge that we have, when we face reality, people buy alcohol on credit. You get money and you have to go pay your alcohol debt. Most of the money ends up in alcohol. Many kids dying from hunger, not necessarily that they die, but because they don’t eat healthy. Parents don’t have time to cook for their kids. We can go now to the townships, we’ll find them [parents] sitting next to taverns, there’s wine next to them.”

-CHW, Nyanga.

“And then there is people, some that is on drugs. Some that is on alcohol. So, I won’t say they can’t afford, but the children don’t get it [food]. They’re using the stuff for their benefits to drink and to have that alcohol and drugs. Then the children is suffering of death because they get the money and they do their stuff, but the children, they all suffer then. That is one of the reasons I also get very ticked about. But what can I do? I can just talk and talk and talk. And they don’t listen to me; you see? It’s very hard like that.”

-CHW, Kensington.

### Lack of individual accountability

Micro-level stakeholders also stressed individual accountability for nutrition. While some social and environmental determinants were recognized, frustration was expressed by some for individuals not taking responsibility for their health and nutrition and for over-relying on government support.

“I think people is just lazy. I’m just lazy. Because, like I said, why must I now waste my time cooking when I can run down the road and just get on my way from work, get something to eat? So, they become lazy.”

-OTL, Kensington.

“They always want government to help them all the time… they don’t want to help them first, they want government to help them. Because when they tell you that there’s no one who’s working at home, they expect you to help them to get their social grant…I think they don’t care about their health… Some of them, they get social grants because they are sick. Government is helping them to go and buy food, good nutrition. But they don’t do that. No, some of them, they just drink that money and default their treatment so that they continue getting the government money. Mhm. They’re doing it on purpose.”

-OTL, Gugulethu.

### Micro-level levers

Micro-level stakeholders recommended two main leverage points to help address the DBM: food gardens and government social support programs such as food packages or grants. 

### Food gardens

Many participants suggested improving the food environment and nutrition in township communities by establishing more backyard and community gardens. Participants reflected on how previous generations relied on growing their own food, making them healthier and able to save money. They noted that land for gardening is limited in the townships but that changes to enable gardening would empower people to eat healthier.

“And if we had more like vegetable gardens… I’m sure that that will alleviate our challenge. We don’t have food, we don’t have this, we don’t have— We have it. It just costs us patience. We must have patience. And at the end of the day, like I said, there must be employment. And adding that community garden as a form of employment for somebody.”

-OTL, Kensington.

“And maybe you will advise by saying we can plant at the back like small gardening at the back. You know veggies, but some of, most of the people, they don’t have space for gardening because they’re living on the squatter camps. So, there is no space for that.”

-OTL, Gugulethu.

### (Government) social support programs

Participants also suggested two means of government support that could improve nutrition: food packages (i.e., parcels) and food welfare payments to those in need. They noted that while welfare payments (i.e., social grants) exist for children, medical needs, and disability, these are not restricted to certain purchases, and food budgets are often not prioritized, linked to the use of alcohol and drugs as noted before. Participants suggested welfare payments dedicated to food with restrictions on the type of spending, or the government directly providing healthy food packages.

“…instead of giving these people money, why can’t they give them food or food stamps? Like they do overseas. It’s like they get SASSA [welfare payments], but they can only go to the shop to buy food stuff. Because other people get that money. And before you know it, there’s no more money left. Because the mother’s thinking, I must get this and this and this for myself. Or I must get my child the name brands whatsoever. And the child will say tomorrow, ‘I’m hungry.’ Because he can’t eat that name brand sweater. There’s no food in the house, so what’s he gonna do?”

-CHW coordinator, Kensington.

“Maybe if government can give them food parcels. I think that will help. I cannot say if the government can give them some social grant [welfare payment], because sometimes what they do, they default, they default their medication because they want such social grant money. Some of them, they don’t even use it to buy food, they buy alcohol and drink alcohol… If maybe the government can give them food parcels, I think that can help.”

-OTL, Gugulethu.

### Meso-level drivers

In the meso-level group, additional drivers related to the DBM included the power of the formal food industry; political inertia; and a siloed government approach.

### Power of formal food industry

Many participants stressed the undue influence of corporate food industries on the South African food environment, with some directly referring to the ‘commercial determinants of health’. Within the interviews, the commercial strategies most identified as contributing to the DBM were marketing, corporate political activities, and the power and influence of large, corporate industries over informal food business.

Firstly, the power of marketing by commercial food actors was strongly identified as affecting the DBM, particularly regarding overweight, obesity, and DR-NCDs. Participants noted that marketing of unhealthy foods through print, social media, and TV was prominent in townships and in Cape Town in general, and that issues with marketing were closely related to food and nutrition literacy. Participants emphasized that many consumers have relatively low food and nutrition literacy and through marketing, companies persuade people that their products are healthy and/or necessary and foster brand loyalty.

“…it’s really important for people to understand how the marketing is really, they’ve completely manipulated people into believing that these products are necessary. I mean everywhere you go… I was looking when we were driving around, every single small shop has Coca-Cola branding. They [the company] come, they bring a fridge in. They do that and they’ve been doing this for years and years and decades now. So, they know exactly what they’re doing; they’re creating brand loyalty.” - Civil society representative.

Secondly, participants described corporate political activities, primarily lobbying, to foster favorable regulations for food industries and to allow corporate food actors to dominate the food environment. One example cited was lobbying by the sugar and beverage industries for the reduction of the sugar-sweetened beverage tax introduced in South Africa in 2018 (the Health Promotion Levy), from the effective tax rate of 20% to 11%. Another example related to agreements with food actors that were made during a recent taxi strike in Cape Town. During these violent strikes, in which 38% of businesses were unable to sustain their daily activities (mostly in low-income areas), the Consumer Goods Council of South Africa signed an agreement with the City of Cape Town to enable police escorts and other safety measures to ensure delivery of their products to markets, while informal retailers were not included in this agreement [26].

“...for me, it’s a representation of the bias towards a particular lobby group. No one was worrying about the informal traders, where most of the people get food. You know, this was a particular lobby group who had an ear of the mayor, who’ve now signed a formal MOU [memorandum of understanding] that when there are other disasters, there is a process in which they will talk to each other. Which just shows how that is unstructured. So how do academics and civil society call out those kind of deals? How do they get wind of them? How do they challenge them? How do they reflect on these as being kind of rather unfair? Because they’re not going to enable food for everybody, they’re going to enable the benefit of those people represented by that lobby group.” -Food systems researcher.

Thirdly, participants reported that some policymakers are not working in the interests of their community, but rather for industry. Participants described the consolidation of the food industry in South Africa, the support of this consolidation and market chain integration by the state, how the industry leverages the accessibility of informal retailers (e.g., spaza shops) to promote primarily unhealthy products, and how these factors resulted in a weakening of the capacities and accessibility of other informal retailers to sell fresh products. There was particular emphasis on the uneven playing field among food retailers.

“We’ve got to recognize that this is not a level playing field and so conditions have been set out by the state that really kind of lay the red carpet down for the big formal actors and sort of policing is in the interests of the big formal actors. And so, when we’re seeing that sometimes the informal sector doesn’t cope as well. Once the big guys come in, we’ve got to recognize that that’s actually also supported by state bias.” -Food systems researcher.

Similarly, a government employee noted:

“Can we think through how we build those spaces so they’re more enabling to the informal food economy? And particularly the parts of the informal food economy that have kind of fresh and nutritious food as their kind of core business. Because what we’re finding is that if you just build it and you just create kind of generic spaces, it’s the formal large retailers who obviously win in any kind of battle there. And so, you just get more KFC’s [Kentucky Fried Chicken] and so on in those spaces. So, you start moving in the wrong direction. And we also know that in terms of the informal economy, it’s incredibly hard to compete on an even playing field between people who are selling T-shirts and people who’re selling fresh fruit and veg, and people who’re selling chips.” -Government employee, Western Cape Government.

Likewise, participants noted the growth of supermarkets and how this has displaced some more localized fresh food markets and how supermarkets are able to ‘undermine the informal sector’ by launching special deals on unhealthy foods at very low costs. Participants noted that the corporate system is highly resistant to change and that there are few examples globally of successful, effective health-oriented regulation of the commercial food industry.

“Global systems as we know them are hugely persistent, you know, and the elites who benefit from them are not motivated to change those systems. And it’s not so much a sort of social statement or a, you know, an activist statement, it’s just a statement of reality. Corporations are very happy doing what they do.” -Government employee, Western Cape Government.

Participants also acknowledged, however, certain benefits of the growth of formal retailers, including improvements in food safety and food access, as aforementioned.

### Political inertia

The second challenge identified by meso-level participants was political inertia or ‘stagnation.’ It was, for example, recognized that South Africa’s current food system is highly resistant to change due to the nature of its design. Participants referred to the legacies of Apartheid, including the dispossession of rural land and farmlands from Blacks to Whites, and the continued state bias towards consolidated, large farms.

“…the unhealthy system we had here hasn’t just happened by consequence. It’s happened by design. So, what needs to be done is kind of thinking of how it’s undesigned. But that’s, you know, we’re dealing with 150 years of deliberate design to create an excluded food system.” -Food systems researcher.

Moreover, participants noted the lack of real incentives for food system change within the current political economy.

“But I mean increasing agency, if you’re not going to address the structural issues, it’s a dead end as well. So, I think it’s got to be top-down, bottom-up and side-in to have any traction. And I think bits and pieces of those things are falling into place in the Cape Town context, but whether they’re all happening at the same time and whether they’re able to withstand the kind of pushback that’s going to come from a system that has no interest in change is where the challenge is.” -Food systems researcher.

In addition, this inertia was also seen as a lack of political will, partly brought on by a fear to make things worse and a scarcity of “real-life” examples on what could work. For instance, one government employee noted that while regulatory action is likely needed at national level to promote healthier food value chains, there are few examples, even from well-resourced countries and communities, on how to best implement policies while balancing potential economic and social tradeoffs:

“You actually have to start getting into the guts of those bigger value chains to understand how to disrupt some of that. And frankly, I don’t think there are many great models around the world about how to do that… we’ve tried to look just to understand what that might look like from other places and even very capacitated cities around the world– New York, Portland, Toronto–you know, places that have some political will behind it as well. There have been kind of sugar taxes… but it’s in the bigger picture against what we’re facing, I think nobody’s really known how to engage in a way that’s tackling the kind of bigger trends around that nutrition transition… And the South African government, I don’t think has the appetite for it. And given the complexity of it, I’m not sure I would be thrilled to, even if there is the interest. I’m not sure; I think we would do more harm than good potentially by disrupting the sector… So, I don’t even know quite what the best-case scenario would look like in that sector.” -Government employee, Western Cape Government.

Others similarly cited the scarcity of good global examples as a challenge.

“I just don’t know how we will get to an understanding between industry and the advocates for good health and good nutrition of how this food system can be changed for the better. Because at the moment, everything that we see in this country, including in the Western Cape and as far and broad as the whole world, the food system is failing us. We have more malnutrition in the world today than ever before. Every country has a problem with malnutrition… Every single country. So, the food system is failing. And it’s failing the most vulnerable people first.” -Nutrition researcher.

It was noted that it requires ‘courageous leadership’ and even ‘activism’ from policymakers to begin to tackle these complex, inter-linked problems around nutrition. The political inertia was closely tied to the challenge of limited resources, as stakeholders discussed the challenge with sustaining momentum for action while juggling many responsibilities and limited funds. For example, a government employee related how after visiting communities, employees came back with a surge of energy to do things differently, but this energy was quickly snuffed out by the everyday requirements of the job:

“…it’s the same old thing with change processes, that everybody [who] went on it is transformed, and then they go back to the office and everybody else looks at them like they’re crazy, like, why are you suddenly making my life harder? Please. *laughs* And so it’s incredibly hard to sustain that energy back at the office, six months later.” -Government employee, Western Cape Government.

### Siloed government

The third major theme identified at meso-level was the siloed approach of government in addressing the DBM across different departments and different tiers of government. There was a consensus that while an intersectoral, transversal, and comprehensive approach to food environment policies is necessary, the default behavior of the current system is to work in silos. Reasons for this included a lack of integration of mandates; lack of platforms for interaction across departments and stakeholders, partially brought about by limited resources; and uncertainty of who is or should be responsible for certain policies.

Firstly, participants noted a lack of cohesion and integration across policy mandates. Within South Africa, legislative mandates are divided among the national, provincial, and local governments. While the right to food is a constitutional right, the different policy areas necessary to ensure a healthy food environment—e.g., nutrition, spatial planning, water and sanitation, education and school feeding—are divided among the different tiers of government and across different departments. This division presents a challenge in trying to build a comprehensive, multi-sectoral response to food environment issues. Furthermore, participants noted these mandates can also be unfunded, which poses an additional challenge for implementation.

“Food and nutrition is not a provincial competency; it’s a national competency. You go to national, they go, no, no, no, it’s a municipal competency. You go to the municipality, they go, well, we don’t have the resources; it’s an unfunded mandate, we don’t get money for this. So, everyone’s got a good reason why it’s not their problem.” -Government employee, Western Cape Government.

Participants highlighted that previous attempts to create a comprehensive food and nutrition strategy have not yet been successful in substantially reducing the DBM. One example was the 2018–2023 National Food and Nutrition Security Plan, which sought to establish an intersectoral National Food and Nutrition Security Council to steer just such an integrated, intersectoral response, but which has not materialized as of January 2025.

“I think it’s 2018 that there’s a new plan that’s produced at a national level that argues for actually a food and nutrition strategy or a plan that brings those things together and starts to talk about systems. In practice, when you actually see how it breaks down, it reintroduces all the silos… because it tries to identify particular targets within mandates of individual departments. So, we’ve just continued to struggle with a very fractured policy environment in South Africa around food and nutrition. Everybody is interested in one piece of the puzzle.” -Government employee, Western Cape Government.

Secondly, participants reported a lack of communication channels between departments and with civil society and academic stakeholders. Participants described scenarios where academic research is not presented in a useful format to policymakers and never utilized for policymaking. A long-time employee of the City of Cape Town described their experience with interdepartmental communication:

“We didn’t speak to each other, that I know… So, in the City in Cape Town… there are different departments that got something within the food systems, within the city… all got the interest in this, but I’m doing my thing because I got a budget to spend. And I’m doing my budget without speaking to another department. And I think that is what happens within the City of Cape Town. And we are not working together.” -Government employee, City of Cape Town.

Finally, a prominent theme was a question of who is or should be responsible for these issues and mandates. As some nutrition-related programs and initiatives are integrated or shared across departments and government levels, there can be ambiguity about who is coordinating and, in the end, held accountable for results. As one nutrition researcher commented, “but you know how the saying goes (…), it’s everybody’s business, but nobody’s real responsibility. So maybe we should rather say, not whose business is it, but whose responsibility is it?”

### Meso-level levers

The meso-level stakeholders suggested several potential leverage points to address the DBM: interventions in the first 1000 days of life, food sensitive urban planning, strengthening networks, and adopting a systems response.

### First 1000 days

One point of priority for participants was a focus on good health and nutrition in the first 1000 days of life (from conception to two years after birth) with an emphasis on maternal and infant health. The Western Cape Government Strategic Framework for Household Food and Nutrition Security (called the Nourish to Flourish Framework) was cited as an innovative and cross-cutting framework within this area that should be continued. Participants noted this leverage point requires intersectoral policies to include various spheres such as maternal health protection (e.g., breastfeeding support and paid maternity leave), early childhood development centers, water and sanitation interventions, and violence reduction.

### Food sensitive urban planning

Another identified leverage area was infrastructure and spatial planning in the urban food environment. Participants noted that the mandate for spatial planning lies at the local level so this is an area that the City of Cape Town can take immediate action to address. Participants suggested improving bylaws regarding land use for gardens, proliferation of fast-food outlets, and stronger infrastructure around informal trader zones. Food sensitive urban planning was particularly valued for its position as an intersectoral subject, bringing together sectors such as economic development, water and sanitation, public health, architecture and urban design, agriculture, and energy, amongst others. Participants suggested education and training within the sectors of public health, architecture, and urban planning should be improved to discuss more transversal issues regarding the impact of urban design on nutrition and the food environment:

“Any kind of food environment intervention needs to understand how the space is used… I think it’s just important to make sure we’re thinking about places as not the spaces where people get food, but as spaces that have all these kind of other functionalities that can either enable or hinder the impact of the intervention.” – Food systems researcher.

One caveat that emerged was that due to the multi-sectoral nature of urban planning, a challenge can be that no department takes final responsibility and initiatives stagnate—further contributing to the siloed approach. This challenge was closely related to the two remaining leverage points of strengthening networks and engaging a systems response.

### Strengthening networks

The importance of strengthening networks was a primary theme, particularly across government sectors and among networks with academia and civil society. While the importance of transparent interactions with industry were mentioned, there was hesitancy about the best way to engage with them in the food sphere (closely related to the discussion on commercial determinants of health).

For one, intragovernmental cross-sectoral networks were highlighted as mitigation for the historically siloed approach of government. Participants also underscored the need for collaboration between government levels and departments (i.e., across provincial and city government). Transparent and routine communication was stressed in order to sustain policy momentum, share information, increase accountability, and ensure a whole-of-government approach.

In addition, participants described the key relationship between academics and government, emphasizing the need for researchers to share understandable and relevant information with policymakers to support governance structures and suggest potential areas of innovation.

Finally, participants recognized the crucial role of civil society and the need for greater collaboration between local organizations, the affected communities, and government. Participants commented that these partnerships help the government to ‘innovate and experiment within communities’ and can facilitate dialogue with affected communities. Partnerships among civil society and government that arose from the COVID-19 pandemic were cited as strong examples of the initiative, goodwill, and speed with which new resources or programs can be developed.

“COVID showed us that things can happen very quickly. Resources can be mobilized. Interesting networks can be utilized. New networks can be built. Deliveries can be made when there’s a cap or a lockdown situation. So, what does that tell us? These things are possible; it’s just not done.” -Nutrition researcher.

### Adopting a systems response

Another identified leverage area was adopting a systems response to the DBM. Stakeholders acknowledged there was no single silver bullet solution to address the DBM, but rather recommended a comprehensive, systemic response with multiple, integrated programs and policies across departments.

“…something like malnutrition, everyone would say that’s the Department of Health’s responsibility when it in fact is not. They can treat it, and they can do some sort of intervention, but in reality, that’s not where it starts, that’s the end point.” –CHW administrator.

In line with strengthening networks, stakeholders emphasized the need for collaboration and mobilization across departments and government levels, and with various stakeholder groups including affected communities and community leaders, civil society, academia, and industry. The importance of routine monitoring and evaluation as part of a systemic response was noted to understand progress and any potential unintended consequences.

Several stakeholders mentioned the newly created Cape Town Resilience Department and the Cape Town Resilience Strategy as a valuable example of a systems thinking approach to food security. Established in 2019 as part of the Rockefeller 100 Resilient Cities Program, the Cape Town Resilience Strategy (and the coordinating Resilience Department) was designed as a roadmap for how the city can plan for and resiliently respond to future shocks and stressors [27]. The Resilience Department has taken on a facilitator role to better coordinate cross-departmental collaboration to meet the objectives of the resilience strategy, one of which is to “establish a food systems programme to improve access to affordable and nutritious food” [27]. However, at least one stakeholder noted that this department is a facilitator and not a strategic planning or implementation department and that a larger budget would be required to follow through on all the objectives.

## Discussion

### Locating key findings in the literature

This study investigated drivers of, and leverage points for addressing the DBM in Cape Town across the micro- and meso-level. Our multi-sectoral stakeholders described four overarching drivers of the DBM: financial constraints, resource constraints, food and nutrition literacy, and the role of informal business. At the micro-level, alcohol and other drug use and lack of individual accountability were described as additional barriers to good nutrition. At the meso-level, challenges included the power of the formal food industry, political inertia, and a siloed government approach. The leverage points across both levels included food gardens, government social support, good health in the first 1,000 days of life, food sensitive urban planning, and adopting a systems response to the DBM.

Our findings are corroborated by other studies, including the 2022 South Africa Food Systems Profile published by the Food and Agriculture Organization (FAO), which included participatory processes to develop a systemic understanding of drivers and levers of a sustainable food system [2]. In this report, income poverty was also identified as a main driver of the DBM [2]. As described by stakeholders in our study, low income was found to contribute to lack of dietary diversity and consumption of less healthy food options [2]. The FAO study also identified multidimensional poverty as an important driver, including access to water, sanitation, and hygiene, food storage, and energy– all resources highlighted by our stakeholders as being limited and fueling the DBM [2]. Erzse et al. (2021) reported similar drivers when examining community perceptions of the factors shaping maternal and child nutrition in the Soweto township, Gauteng, South Africa. Poverty was also highlighted as the greatest barrier to optimal nutrition, with food safety of informal food businesses and substance abuse named as prominent concerns [31]. Nutrition knowledge and skills were stressed as an important solution to improve community nutrition as were school- and home-based gardens and social grants specific to food purchases [31].

At the micro-level, the frustration expressed by some stakeholders regarding individual lack of accountability for nutrition and the emphasis on ‘laziness’ and over reliance on government aid relates to the common discussion of welfare dependency in South Africa. As described by Pettersson (2022), in South Africa there is a “prominent fear that social grants feed a negative dependency and an unsustainable mindset of expecting things for free, undermining people’s incentives to work, save, or invest…” [32]. On the other hand, research by scholars and prominent South African and international organizations have found that welfare payments in South Africa reduce poverty and inequality, increase purchasing power, improve nutrition among children, and promote labor market participation [33–36]. The current system of welfare payments has an extensive reach with about one in three South Africans being “a direct beneficiary of a social grant, while nearly two-thirds of the population are either direct or indirect beneficiaries of the system” [36]. However, recent public hearings have reported abuse of payments to pay for items like alcohol and gambling, as described in this study [37]. The identified leverage point of welfare benefits explicitly for food purchases (i.e., food vouchers), similar to the Supplemental Nutrition Assistance Program (SNAP) in the United States or the Food Scholarship for Higher Education Programme in Chile, could, therefore, potentially reach large segments of the most vulnerable populations while reducing misuse of social grant funding [38].

The emphasis on personal accountability for nutrition can also be understood within the context of the commercial determinants of health as underscoring individual responsibility is a standard narrative deployed by the corporate food industry as a strategy to avoid regulation and shape public opinion [39, 40]. It is important, therefore, to understand individual behavior change as a necessary element to improve nutrition, but within the larger context of creating an enabling food environment to support healthy food choices and improve agency. Recently recognized by the FAO as one of the six dimensions of food security, agency “implies the capacity of individuals or groups to make their own decisions about what foods they eat, what foods they produce, how that food is produced, processed and distributed within food systems, and their ability to engage in processes that shape food system policies and governance” [41]. Improved food and nutrition literacy (including recognition of the breadth of the overall food environment), as well as an increase in food gardens, may help improve agency and shift the narrative of one focused solely on individual accountability to one that recognizes the individual within the context of the personal and the external food environment [42]. In addition to promoting agency, food gardens have been noted to contribute to greater social inclusion of women and to support household food security through direct consumption of the products, savings on household food expenditures, and through income generation via market participation with excess crops [43].

At the meso-level, the challenges found in our study are consistent with other studies examining the role of governance in creating enabling healthy food environments in low- and middle-income contexts. An analysis of political challenges in addressing the DBM in nine countries of Southeast Asia and the Pacific highlighted the lack of a “single pathway to good nutrition” and the need for a whole-of-government and systemic response [44]. The authors also noted the lack of successful models, even from high-resourced contexts, on what is effective to reduce overnutrition and a weak evidence base from the countries experiencing the DBM, including “holes in malnutrition situation data” and credible impact assessments of interventions [44]. Our stakeholders equally stressed the need for stronger monitoring and evaluation of food system interventions and nutrition indicators.

Additionally, the recognition of the effects of corporate actors on health has grown in prominence in the field of public health over the past decade, and the ultra-processed food industry is recognized among the four primary unhealthy commodity industries contributing to at least a third of global preventable deaths each year [45, 46]. In the *Lancet* series on the commercial determinants of health, the politics around sugar-sweetened beverage consumption in South Africa illustrates the influential corporate tactics used to ‘weaken and delay’ regulations like the sugar tax and front-of-pack labeling [46]. In our study, the intense marketing and lobbying of the formal food industry was reported to strongly influence the food environment. Lack of policy coherence and hesitancy on how or whether to engage in partnerships with industry have also been highlighted as a challenge by our stakeholders, as in the *Lancet* series [46].

The 2020 *Lancet* series on the DBM, meanwhile, identified several double-duty actions to address the DBM, which were echoed by our stakeholder groups, including prioritizing nutrition and health in the first thousand days; conditional cash transfer or food transfer programs (with a strong health education component); improving the food environment through more urban and school gardens; and, reducing the availability of unhealthy processed foods [47]. Food sensitive urban planning, another leverage area suggested by our stakeholders, offers an approach not only to reduce the availability of unhealthy processed foods, but has also been noted for its potential to alleviate the underlying elements of multidimensional poverty that hinder good nutrition: improved infrastructure enables sustainable water and sanitation in informal settlements and townships [48]; improved public transportation facilitates food accessibility and reduces commute times; and improved household energy (i.e., electricity and fuel) access enables cooking and storage of foods at home [4], to name a few examples. At the same time, food sensitive urban planning explicitly recognizes and includes the informal sector as an important actor in the food system, which promotes social inclusion of primarily the urban poor [49].

The DBM in South Africa is a crisis born from a complex adaptive system. As in other health crises, it needs to be addressed by layers of action with both short-term responses to immediate needs and long-term structural, systemic change based on recognition of the social determinants of health. Creating an enabling food environment through more coherent intersectoral governance is necessary for sustainable change to reduce the DBM and improve all dimensions of food security.

### Strengths and limitations

A key strength of our study was the engagement of a broad variety of stakeholders with a diversity of gender, ethnicity, and area of work and expertise. By engaging with micro- and meso-level stakeholders, we were better able to probe the various challenges and levers and explore the realities of those affected by the DBM and those working in food environments. The inclusion of three township study sites likewise provided a greater range of perspectives, particularly as one site (Kensington) varies more sharply in ethnic and socio-economic demographics compared to the other two sites. Another strength was the diversity of the research team, which helped to make our work more inclusive for stakeholders with different languages and cultural backgrounds. By interviewing people in English and the main local language (isiXhosa), we were able to include a wider diversity of participants. It is possible, however, that we failed to capture some nuances in the isiXhosa language during analysis. Another limitation was that primary data analysis was conducted by one researcher from the U.S.; however, the researcher was able to spend three months in Cape Town to better understand the context, and the collaborative group coding and manuscript editing with partners from South Africa helped to ensure more insider perspectives and cultural specificities were captured. Moreover, while we strove to include a range of sectors at the meso-level, the study could have benefitted from participation of more stakeholders from Departments such as water and sanitation, urban mobility, or urban planning and design. The lack of representation from informal business is another limitation. As the importance of these stakeholders was emphasized in this study, future research should include their perspectives on challenges and possible levers. A final limitation is the overwhelming presence of women in the micro-level interviews. Women work more often as CHWs and are often responsible for food preparation in households, hence may be considered representative of community perceptions. Future research could include more men at the micro-level as well as children, teenagers, and young adults, who are also significantly affected by the DBM, highly targeted by food marketing, and often less consulted on their perspectives.

## Conclusions

This study showed how the DBM affects the development and potential of individuals and communities in Cape Town. While new strategies and commitments have attempted to create a more systemic response to the DBM, it is necessary to address the issue with a systems lens and to meaningfully and actively engage with affected communities to develop solutions. This study identified several micro- and meso-level determinants that drive the DBM in this setting and provides evidence of the need for structural changes supported by food sensitive policies across different socio-ecological levels to optimize nutrition.

## Supplementary Information

Supplementary Material 1.

## Data Availability

The interview data are not publicly available due to the sensitive nature of the data that could compromise the privacy of research participants. The codebook and additional information are provided in the supplementary material.

## Abbreviations

CHW: Community health worker
DBM: Double burden of malnutrition
FAO: Food and Agriculture Organization
NGO: Non-governmental organization
OTL: Outreach team leader
WHO: World Health Organization
