# Supplementary material for: Drivers and levers of the double burden of malnutrition in Cape Town, South Africa: insights from in-depth interviews with multi-sectoral stakeholders
Source: BMC Public Health. 2025 Aug 29;25:2966. doi: 10.1186/s12889-025-24210-0 (PMC12395704; doi:10.1186/s12889-025-24210-0)
Supplement: Supplementary file 1 — Supplementary Material 1. [file 12889_2025_24210_MOESM1_ESM.docx]

**Supplementary material**

**Supplementary material to:** Nicole Holliday, Mulalo Kenneth Muhali, Martina Lembani, Hlolisiso Nonkeneza, Maxwell Feni, Zandile June-Rose Mchiza, Jillian Hill, Carmen Klinger, Eva A Rehfuess, Peter von Philipsborn, Peter Delobelle (2025): Drivers and levers of the double burden of malnutrition in Cape Town, South Africa: insights from in-depth interviews with multi-sectoral stakeholders

**Corresponding author**: Nicole Holliday, Chair of Public Health and Health Services Research, LMU Munich, [Nicole.holliday@ibe.med.uni-muenchen.de](mailto:Nicole.holliday@ibe.med.uni-muenchen.de)

**Table of Contents**

1. Literature review ………………………………………………………………………….. 1
2. Reporting guideline ………………………………………………………………………. 4
3. Interview topic guide …………………………………………………………………….. 6
4. Codebook ………………………………………………………………………………………. 8

# **Literature review**

*Framing Selection*

To inform the development of the interview topic guide and select the appropriate framing for the societal level at which to focus the interviews (macro-, meso-, or micro-level), we conducted a literature search to identify the existing knowledge base on the drivers and outcomes of the double burden of malnutrition (DBM) in South Africa, how this work had been conducted (e.g., if any participatory methods had been used), and any knowledge gaps. We used Google Search and Google Scholar to identify reports, policy briefs, and government documents and used PubMed to search for relevant journal articles. We reviewed 22 articles including policy briefs, reports, journal articles, and conference papers and discussions (from research institutes and organizations like the WHO and FAO). Primary focus was given to literature from the South African context. Most of the included literature was based on observational studies, outputs of symposiums or conferences, or expert opinion and experience verified with data.

The main finding regarding drivers of the DBM was that drivers occurred at all levels of the system (macro-, meso-, and micro-level), but that research was primarily focused at the structural, macro-level causes. The main drivers identified in the literature could be grouped into the five following themes: urbanization and economic development, poverty and income inequality, lack of sustained change, lifestyle factors, and cultural/societal expectations. All these drivers in turn affected the food environments. A more detailed description of the identified drivers is provided in Figure 1. It should be noted that all figures are in a preliminary form and do not reflect all the intricacies and connections between the various factors.


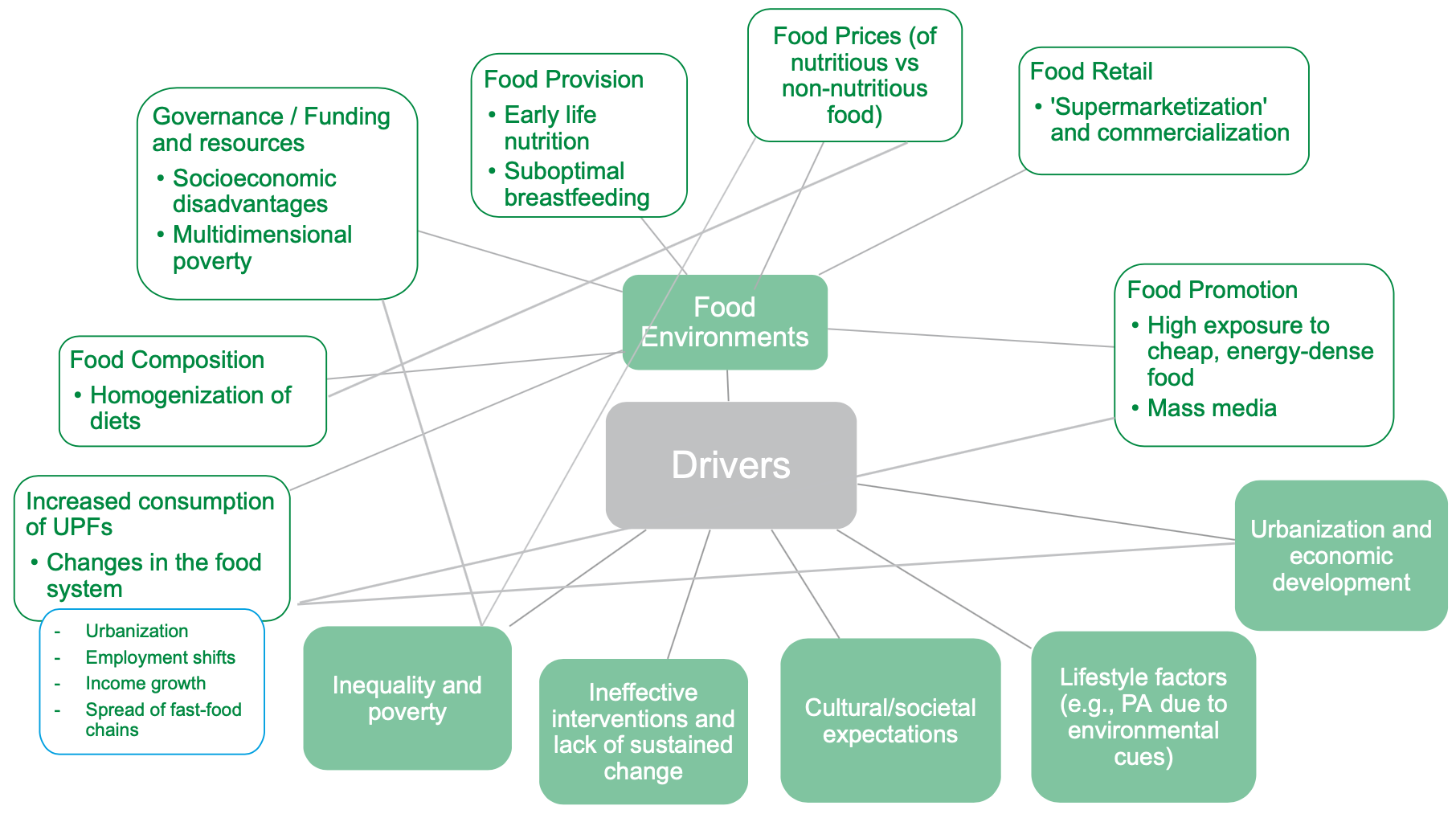


Figure 1. Drivers of the DBM identified from literature search. Note, the lines do not show weight or direction.

Regarding the levers to address the DBM, the literature highlighted the key point that the targeting of just one lever would not be sufficient to address the complexity of the DBM. There were three main categories of levers identified, each of which consisted of several recommended policies and/or programs that would need to be implemented in order to utilize the lever: food environments, interventions, and inequality and poverty. A more detailed description of the identified levers is provided in Figure 2.


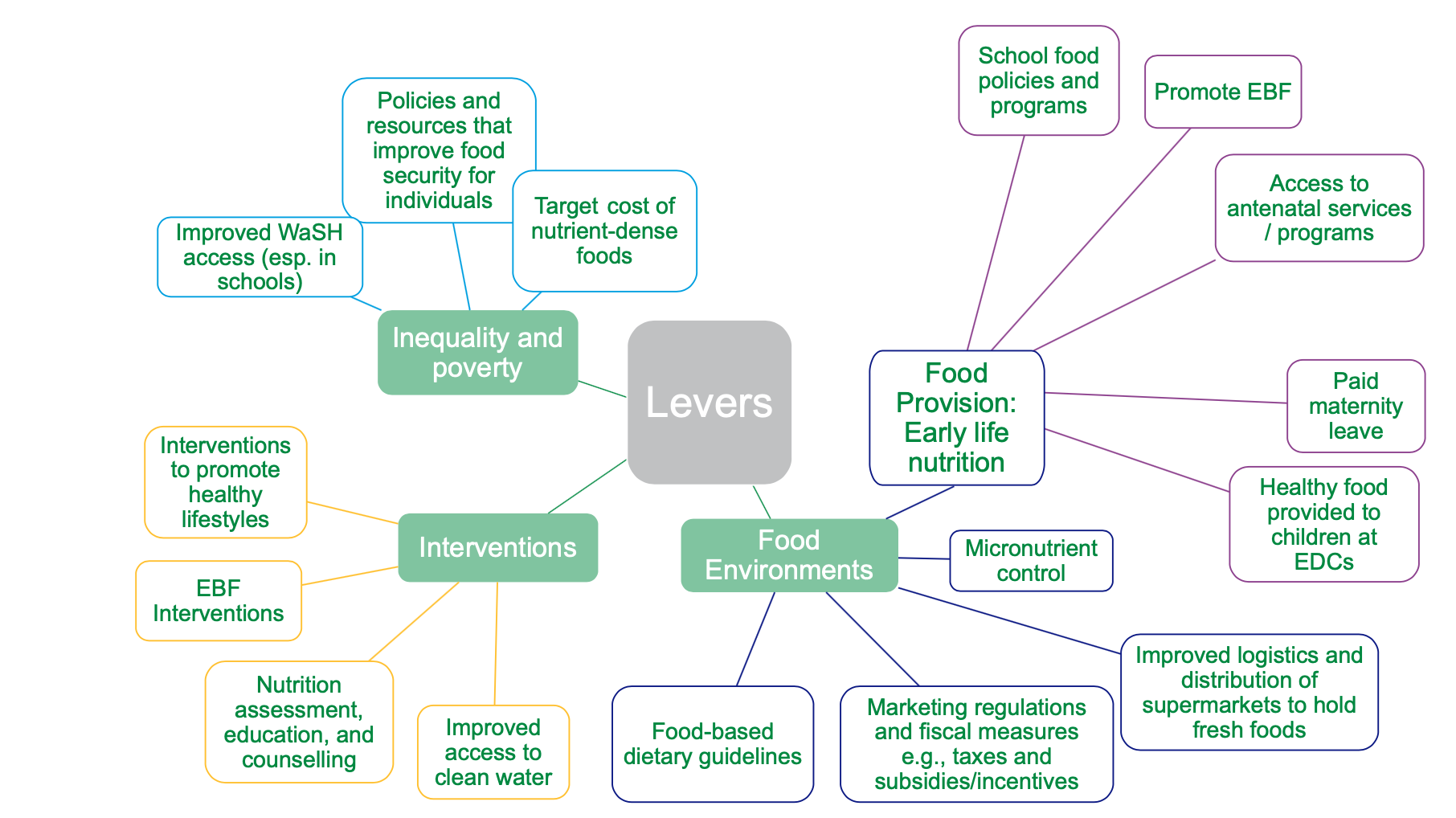


Figure 2. Levers to address the DBM identified from literature search. Note, the lines do not show weight or direction.

One particularly relevant publication is the 2022 policy brief “Food Systems Profile—South Africa,” published by the Food and Agriculture Organization of the United Nations (FAO), the French Agricultural Research Centre for International Development (CIRAD), and the European Union. This rapid assessment of the food system in South Africa combined both qualitative and quantitative data and included participatory approaches, including four thematic workshops with stakeholders from public, private, and society sectors. The report identified four challenges of the South African food system and within each of the challenges, further analyzed the drivers, levers, and outcomes of each challenge. The four challenges included: 1) improved nutrition; 2) sustainable agricultural production systems; 3) levelling the food system playing field; and 4) improved food system governance.

As this report was similar in both framing (i.e., focusing on systemic challenges in the food system) and approach (i.e., participatory methods with diverse stakeholders) to our current research, we decided to focus our work on the meso- and micro-level drivers and outcomes as opposed to the macro-level focus utilized in the FAO report.

1. **Reporting guideline**

The *Consolidated Criteria for Reporting Qualitative Research* (COREQ) checklist is shown below. Page numbers with a preceding s (e.g. s3, s5) refer to the supplementary material. The checklist is reproduced and adapted with permission from Tong *et al.,* 2007.

*Indicates where an adaptation was made to the original checklist

N/R = not reported

| **Item** | **Guiding questions/description** | **Page number** |
| --- | --- | --- |
| **Domain 1: Research team and reﬂexivity** | | |
| ***Personal Characteristics*** | | |
| 1. Inter viewer/facilitator | Which author/s conducted the interview or focus group? | 8-10 |
| 2. Credentials | What were the researcher’s credentials? E.g. PhD, MD | N/R |
| 3. Occupation | What was their occupation at the time of the study? | 8-10 |
| 4. Gender | Was the researcher male or female?  *What are the genders of the research team? | 8-10 |
| 5. Experience and training | What experience or training did the researcher have? | 8-10 |
| ***Relationship with participants*** | | |
| 6. Relationship established | Was a relationship established prior to study commencement? | 7-8 |
| 7. Participant knowledge of the interviewer | What did the participants know about the researcher? e.g. personal goals, reasons for doing the research | N/R |
| 8. Interviewer characteristics | What characteristics were reported about the interviewer/facilitator? e.g. Bias, assumptions, reasons and interests in the research topic | N/R |
| **Domain 2: study design** | | |
| ***Theoretical framework*** | | |
| 9. Methodological orientation and Theory | What methodological orientation was stated to underpin the study? e.g. grounded theory, discourse analysis, ethnography, phenomenology, content analysis | 9 |
| ***Participant selection*** | | |
| 10. Sampling | How were participants selected? e.g. purposive, convenience, consecutive, snowball | 7-8 |
| 11. Method of approach | How were participants approached? e.g. face-to-face, telephone, mail, email | 7-8 |
| 12. Sample size | How many participants were in the study? | 11 |
| 13. Non-participation | How many people refused to participate or dropped out? Reasons? | N/A |
| ***Setting*** | | |
| 14. Setting of data collection | Where was the data collected? e.g. home, clinic, workplace | 9 |
| 15. Presence of non-participants | Was anyone else present besides the participants and researchers? | 9 |
| 16. Description of sample | What are the important characteristics of the sample? e.g. demographic data, date | 11 |
| ***Data collection*** | | |
| 17. Interview guide | Were questions, prompts, guides provided by the authors? Was it pilot tested? | s5-6 |
| 18. Repeat interviews | Were repeat interviews carried out? If yes, how many? | N/A |
| 19. Audio/visual recording | Did the research use audio or visual recording to collect the data? | 9 |
| 20. Field notes | Were ﬁeld notes made during and/or after the interview or focus group? | 10 |
| 21. Duration | What was the duration of the interviews or focus group? | 11 |
| 22. Data saturation | Was data saturation discussed? | 8 |
| 23. Transcripts returned | Were transcripts returned to participants for comment and/or correction? | N/R |
| **Domain 3: analysis and ﬁndings** | | |
| ***Data analysis*** | | |
| 24. Number of data coders | How many data coders coded the data? | 8-9 |
| 25. Description of the coding tree | Did authors provide a description of the coding tree? | s8-14 |
| 26. Derivation of themes | Were themes identiﬁed in advance or derived from the data? | 9 |
| 27. Software | What software, if applicable, was used to manage the data? | 10 |
| 28. Participant checking | Did participants provide feedback on the ﬁndings? | N/R |
| ***Reporting*** | | |
| 29. Quotations presented | Were participant quotations presented to illustrate the themes/ﬁndings? Was each quotation identiﬁed? e.g. participant number | 12-35 |
| 30. Data and ﬁndings consistent | Was there consistency between the data presented and the ﬁndings? | 12-40 |
| 31. Clarity of major themes | Were major themes clearly presented in the ﬁndings? | 12 |
| 32. Clarity of minor themes | Is there a description of diverse cases or discussion of minor themes? | N/R |

# **Interview Topic Guide**

#
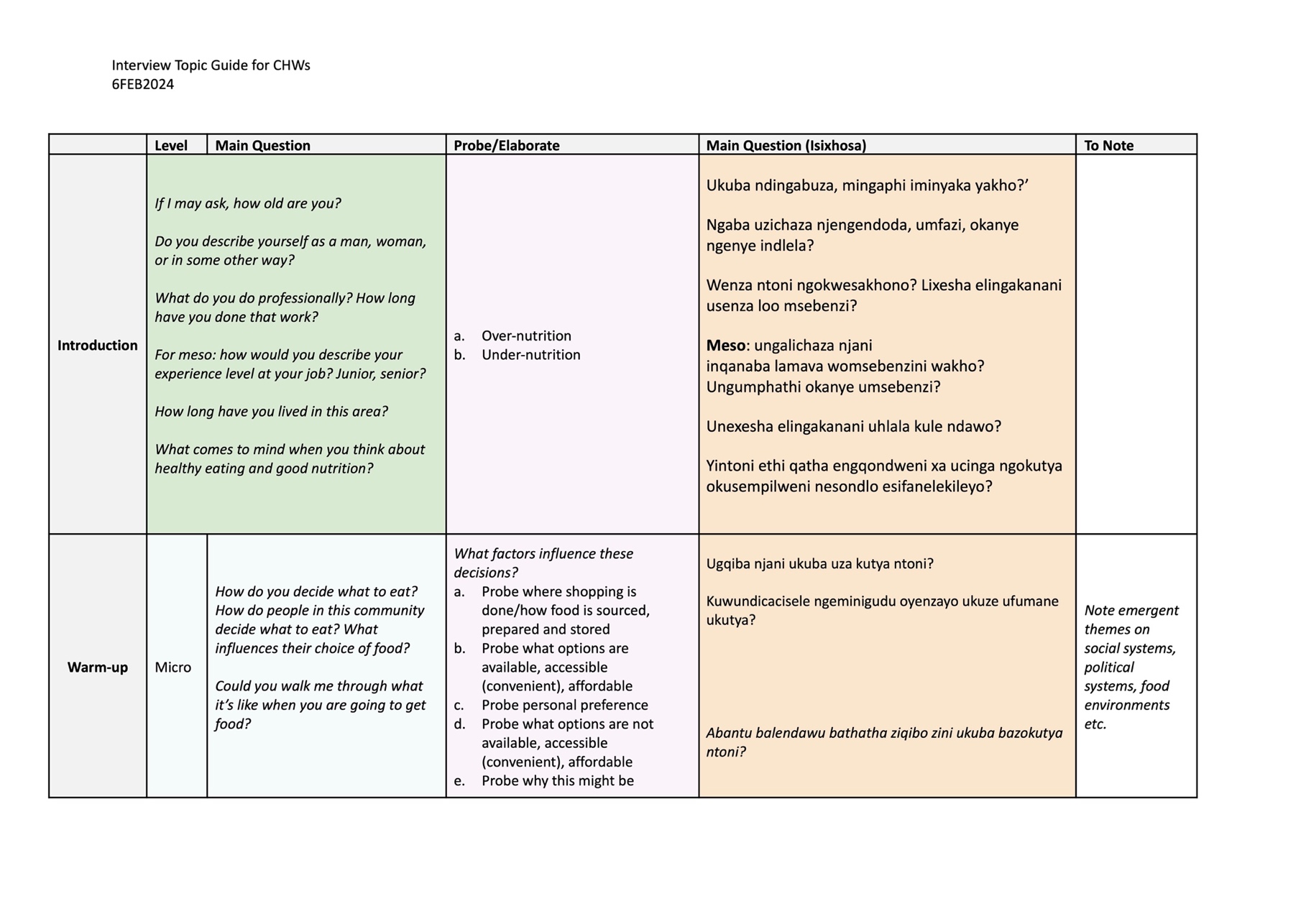


**
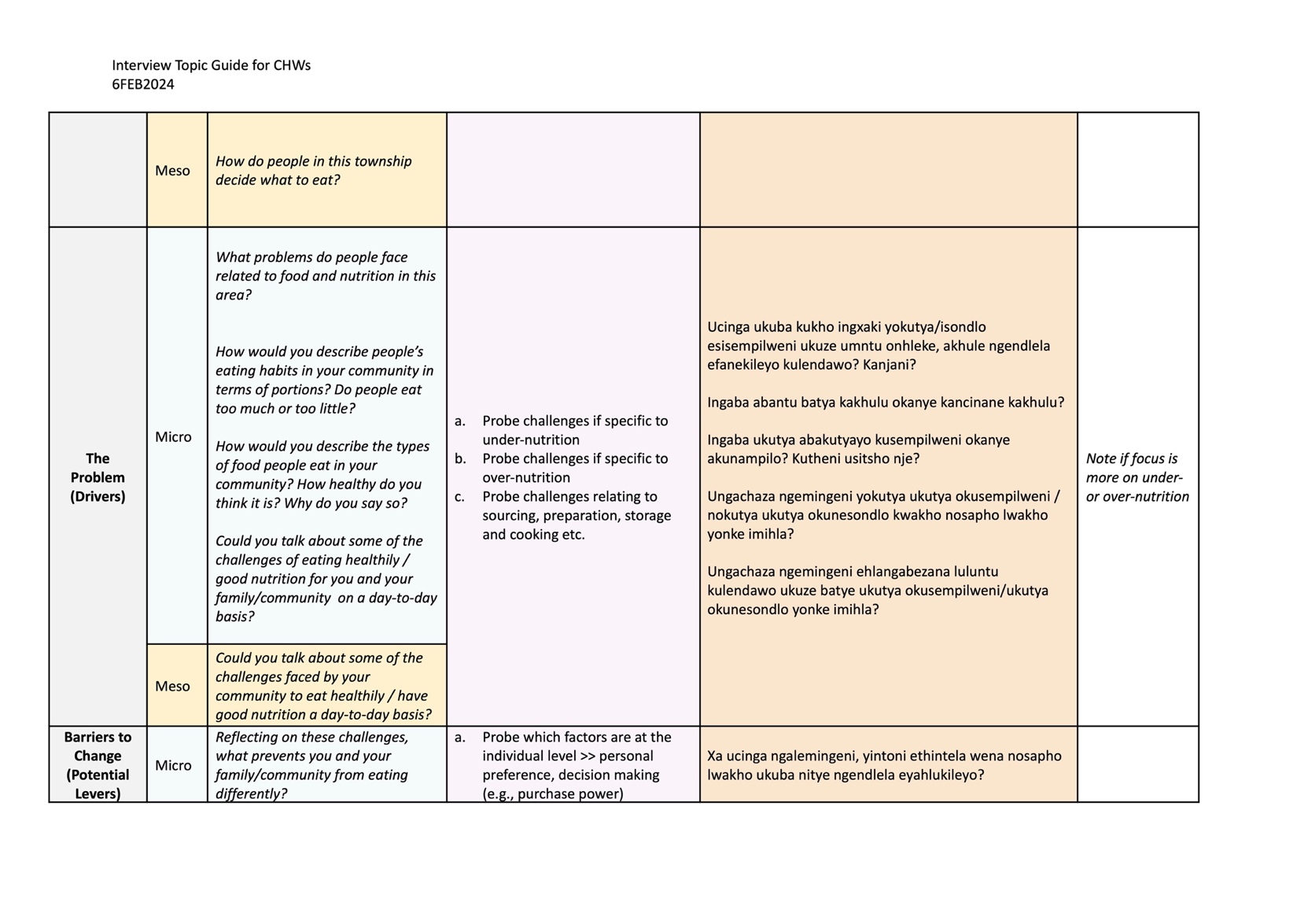
**

**
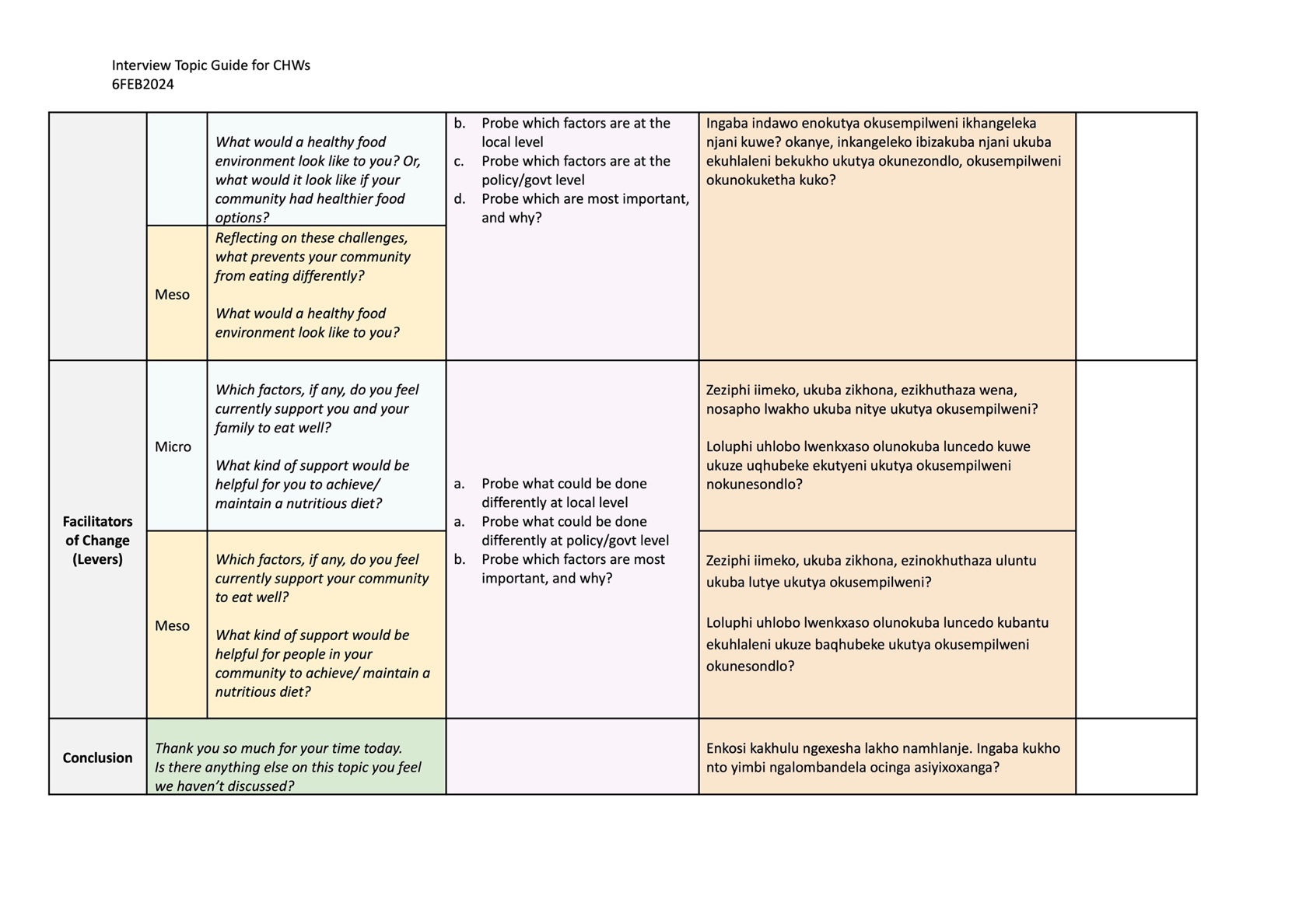
**

**
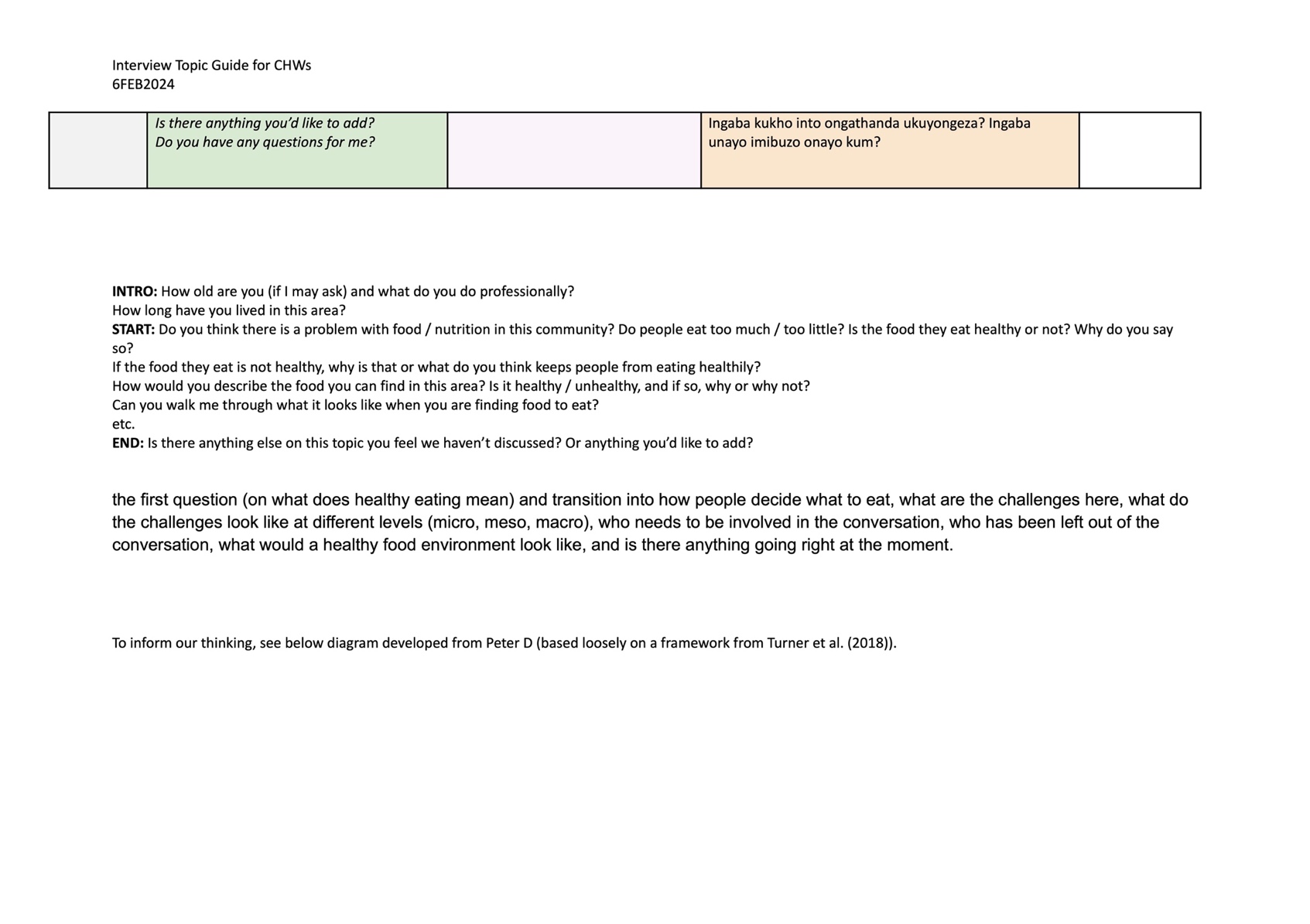
**

1. **Codebook**

| **Meso-Level Interview Codes** | |
| --- | --- |
| **Broad Theme: Resources** | |
| **Code family** | **Definition** |
| Resources_water | Discussion of water as a resource/commodity (includes drinking water, water for irrigation). If referring to water for WASH, can also be coded under CC_WASH. |
| Resources_energy | Discussion of energy as a resource. Includes loadshedding, electricity, oil. |
| Resources_constrained | Discussion of the limitations of various resources. Can include tangible and intangible resources (e.g., water, staff, capacity, money). |
| Resources_funding | Discussion of funding for government/public services. Includes funding for staff positions, funding for social grants. |
| Resources_time | Discussion of time as a resource. Includes scarcity of time, time poverty, limited time. Can also be coded under Resources_constrained, if relevant. |
| Resources_innovation | Discussion of innovation or other resources perhaps not traditionally thought of (e.g., people) that can be utilized. |
| Resources_business | Discussion of ways that industry/private sector (outside of food industry) can support nutrition/food environments. |
| **Broad Theme: Food industry** | |
| **Code family** | **Definitions** |
| FI_food marketing | Discussion of marketing, advertising of foods/drinks. Includes physical media (posters, ads) and media like TV commercials, radio ads. Also includes social media, which is also coded under SP_social media. |
| FI_brand trust | Discussion of trust of food industry brands. Includes brand loyalty, building trust, breaking trust, methods of creating trust, outcomes of brand trust. |
| FI_informal market shaped by formal industry | Discussion of ways or methods by which the informal food market (e.g., street food vendors) is influenced by the formal food industry (e.g., in policy, tactics, reach). |
| FI_informal market | Discussion of the informal food market. Includes spaza shops, corner stores, informal traders. |
| FI_industry influence in policy | Discussion of ways/manner in which the food industry (formal or informal) shapes government policy (at any level). This may include lobbying, sponsorship, funding research, etc. May also be coded under FI_commercial determinants, if relevant. |
| FI_reformulation | Discussion of food reformulation. |
| FI_industry as partners | Discussion of partnerships between government/NGOs and industry. This can be best-practice examples, strategies, or critiques. |
| FI_commercial determinants | Discussion or examples of the commercial determinants of health. |
| **Broad Theme:**  **Social determinants of health** | |
| **Code family** | **Definitions** |
| SD_equity | Discussion of equity, inclusion. This includes discussion of vulnerable groups. |
| SD_power imbalance | Discussion of income inequality, consolidation of power, tactics for holding onto power, ways to balance power, collusion. |
| SD_social grants | Discussion of any social grants (includes food parcels), grants provided to consumers by the government. |
| SD_poverty | Discussion related to poverty, income, wealth. |
| SD_employment | Discussion related to jobs, job loss, unemployment, or job opportunities. |
| SD_debt | Discussion related to financial debts. |
| SD_housing | Discussion related to housing, both formal and informal. |
| SD_apartheid | Discussion related to the history of South Africa, includes discussion of apartheid and legacy or impact of apartheid. |
| SD_advocacy | Discussion related to advocacy (the need for it, how to do it, its role). Includes discussion on integrated knowledge translation with policymakers/stakeholders, where relevant. |
| **Broad Theme:**  **Social perceptions** | |
| **Code family** | **Definitions** |
| SP_framing of food | Discussion of how food is framed in policy and public discourse. Includes discussion of food justice, food sovereignty, right to food. |
| SP_stigma | Discussion of stigmas or normalizations related to food/diet/nutrition culture. |
| SP_food taste | Discussion of the taste of food (likability, acceptability, preference). |
| SP_food culture | Discussion of the cultural aspects around food. Includes local, regional, national food culture. |
| SP_consumer resistance to change | Discussion of consumers being resistant to changes in their diet/dietary behavior. Includes discussion of why consumers are resistant to change. |
| SP_public discourse | Discussion of public discourse (i.e., social narratives) related to food/food environments/nutrition, whose role is it, what are the perceptions of responsibility, etc. This includes public sentiments on healthy diets/DBM/nutrition. Can also be coded as stigma, when relevant. |
| SP_social media | Discussion of social media. |
| SP_consumer perception | Discusses perceptions of consumers regarding foods/what is a healthy diet. |
| SP_individual choice | Discussion of an individual’s ability or right to choose foods. |
| SP_public participation | Discussion of public participation in democracy/governance. |
| **Broad Theme:**  **Governance** | |
| **Code family** | **Definitions** |
| Govern_taxation | Discussion of taxes, tax policy. Includes Health Promotion Levy. |
| Govern_political will | Discussion of political will (i.e., the determination of a political actor to do/say things to produce a desired outcome.) This can include a lack of political will, barriers to political will, corruption, etc. |
| Govern_champions | Discussion of champions (i.e., individuals, particularly political actors, who promote/advocate for health-related causes among their peers). |
| Govern_sustainability | Discussion of the long-term pressure/engagement for change. This can include a lack of long-term commitment or ability. Includes burnout. |
| Govern_learning what works | Discussion of the need for/the experience of learning what works from past experience or other examples. Discussion also of lack of examples to learn from. |
| Govern_who’s responsible | Discussion of who has ownership over policies/programs, who has authority, who has mandates, how decisions are made about who is responsible for implementation, coordinator, monitoring and evaluation, etc. Includes discussion on compliance. |
| Govern_monitoring and evaluation | Discussion of monitoring and evaluation of policies/programs. Includes challenges, value, methods, extent. Includes discussion of data and research. |
| Govern_political economy | Discussion of political economy (i.e., the relationship between individuals, government, and public policy or put another way, how politics effects the economy and how the economy in turn effects politics). |
| Govern_food sensitive planning | Discussion of planning (e.g., urban planning) that consciously considers effects on/relation to the food system. |
| Govern_scales | Discussion of how programs/policies can be scaled to target larger populations. |
| Govern_role of government | Discussion of the various roles of government. Includes all levels of government. This also includes discussion on the different spheres of government, government transparency. |
| Govern_policy framing | Discussion of how policies are framed. |
| Govern_mentality | Discussion of the mental models/mentality of policymakers/government officials regarding food environments, nutrition, or governance in general. Includes discussion of priorities. |
| Govern_litigation | Discussion of court cases/litigation. |
| **Broad Theme:**  **Physical environment** | |
| **Code family** | **Definitions** |
| Physical_food environment | Discussions specifically referring to food environments (i.e., the physical, social, economic, and political context in which people access food). |
| Physical_infrastructure | Discusses physical infrastructure. |
| **Broad Theme:**  **Networks** | |
| **Code family** | **Definitions** |
| Networks_strengthening networks | Discusses the role or value of creating and/or strengthening networks and partnerships between different organizations or individuals. Discussion of networks across different departments/sectors can be coded under this AS WELL AS under cross-cutting issue. |
| Networks_civil society | Discusses the roles, impact, use, etc. of civil society such as NGOs. |
| Networks_academic partners | Discusses the roles, impact, use, etc. of academic/research persons, groups, or institutions. |
| Networks_organizational change | Discussion of change within organizations, the need for change, how to change, elements of change, etc. |
| **Broad Theme: Double burden of malnutrition** | |
| **Code family** | **Definitions** |
| DBM_food insecurity | Discusses food insecurity, hunger, or undernutrition. Includes micronutrient deficiencies. |
| DBM_obesity | Discusses overweight, obesity, or obesogenic environments. |
| DBM_stunting | Discusses stunting. |
| **Broad Theme:** **Food** | |
| **Code family** | **Definitions** |
| Food_access | Discusses issues related to food access. This can include accessibility related to transportation, availability in the environment, time, knowledge of healthy foods. |
| Food_affordability | Discusses issues related to the affordability/cost of food. |
| Food_storage and preparation | Issues related to the storage of food or the preparation (such as cooking) of food. |
| Food_safety | Discusses issues of food safety or foodborne illnesses. |
| **Broad Theme: Cross-cutting issue** | |
| **Code family** | **Definitions** |
| CC_WASH | Discusses issues related to water, sanitation, and hygiene. This includes WASH infrastructure. |
| CC_schools | Discusses schools of any level (not ECDs). |
| CC_ECDs | Discusses early child development centers. |
| CC_agriculture | Includes mentions of agriculture, agroecology, peri urban agriculture, or food gardens. |
| CC_health/nutrition literacy | Includes discussion of health or nutrition literacy (i.e., consumers’ health knowledge, what is a healthy diet, etc.) |
| CC_training and education | Includes discussion of training or education programs for groups such as policymakers, medical providers, urban planners, or food service staff, for example. If it regards health education for the general population/consumers, this would fall under health/nutrition literacy. |
| CC_public health programs | Discussion of health programs such as deworming or immunizations. Does not include WASH programs. |
| CC_safety and security | Discussion of security (i.e., personal security, crime). Includes discussion of police. |
| CC_collaboration | Discussion of the value, role, or challenges of cross-sectoral collaboration. Includes discussion of silos. |
| **Broad Theme:**  **Maternal and child health** | |
| **Code family** | **Definitions** |
| Maternal_infant and young child feeding | Discusses foods and feeding for infants and young children. Includes breastfeeding. |
| Maternal_first thousand days | Discusses the first thousand days of life related to the health of children and mothers. |
| **Broad Theme:**  **Role of cities** | |
| **Code family** | **Definitions** |
| City_urbanization | Discussion of urbanization, the role of the urban environment (i.e., cities) in the food system/food environment. |
| City_geographic diversity | Discussion of the difference/diversity between geographic locations. |
| City_local context | Discussion of issues related to understanding the local context/dynamics. This also includes discussion on empowering locals to be the experts. |
| **Broad Theme: Complexity** | |
| **Code family** | **Definitions** |
| Complex_systematic approach | Discussion of systematic/complex problems or approaches to solutions. This is similar to cross-cutting issues but would also include more general discussion of the need for a multi-arm or multi-sector response. Includes discussion of intergenerational issues. |
| Complex_comprehensive response | Discussion of the need for or how to have a comprehensive response to the DBM/population nutrition. |
| Complex_trade-offs | Discussion of negative externalities or considerations that must be made when implementing programs/policies. Discussion of incentives vs. regulations. |

| **Micro-Level Interview Codes** | |
| --- | --- |
| **Code family** | **Definitions** |
| Micro_individual responsibility | Discussion of an individual’s own accountability for their nutrition/health. Includes discussion of laziness. |
| Micro_gardens | Discussion of individual or community gardens. |
| Micro_spaza shops | Discussion of spaza shops and other informal traders. |
| Micro_food and nutrition literacy | Discussion of food and nutrition literacy or education. |
| Micro barriers_alcohol and drugs | Discussion of alcohol or drug use. |
| Micro_social grants | Discussion of social grant funding/use/misuse. |
| Micro_poverty | Discussion of individual or community poverty/finances. |
| Micro_safety | Discussion of issues related to safety. Includes discussion of crime, feelings of safety. |
| Micro_hunger | Discussion of hunger or lack of food. |
| Micro_transportation | Discussion of issues related to transportation. Includes discussion of modes of transportation, challenges of transportation, or suggestions for transportation. |
| Micro_community resources | Discussion of community resources to improve health and nutrition. |
| Micro_employment | Discussion of employment issues, including unemployment. |
| Micro_food safety | Discussion of food quality, including food safety (e.g., expired food, bugs, foodborne illness). |
| Micro_food affordability | Discussion of the cost of food. |
| Micro_generational | Discussion of generational differences/similarities. |
